# Possibilities and limitations of solution-state NMR spectroscopy to analyze the ligand shell of ultrasmall metal nanoparticles

**DOI:** 10.1039/d4na00139g

**Published:** 2024-05-31

**Authors:** Natalie Wolff, Christine Beuck, Torsten Schaller, Matthias Epple

**Affiliations:** a Inorganic Chemistry, Centre for Nanointegration Duisburg-Essen (CeNIDE), University of Duisburg-Essen 45117 Essen Germany matthias.epple@uni-due.de; b Structural and Medicinal Biochemistry, Centre for Medical Biotechnology (ZMB), University of Duisburg-Essen 45117 Essen Germany; c Organic Chemistry, University of Duisburg-Essen 45117 Essen Germany

## Abstract

Ultrasmall nanoparticles have a diameter between 1 and 3 nm at the border between nanoparticles and large molecules. Usually, their core consists of a metal, and the shell of a capping ligand with sulfur or phosphorus as binding atoms. While the core structure can be probed by electron microscopy, electron and powder diffraction, and single-crystal structure analysis for atom-sharp clusters, it is more difficult to analyze the ligand shell. In contrast to larger nanoparticles, ultrasmall nanoparticles cause only a moderate distortion of the NMR signal, making NMR spectroscopy a qualitative as well as a quantitative probe to assess the nature of the ligand shell. The application of isotope-labelled ligands and of two-dimensional NMR techniques can give deeper insight into ligand-nanoparticle interactions. Applications of one- and two-dimensional NMR spectroscopy to analyze ultrasmall nanoparticles are presented with suitable examples, including a critical discussion of the limitations of NMR spectroscopy on nanoparticles.

## Abstract 2

Unlike larger plasmonic nanoparticles, ultrasmall nanoparticles with a diameter of 1–2 nm can be well analyzed by NMR spectroscopy. This gives deep insight into the nature of the organic ligand shell.

## Graphical abstract

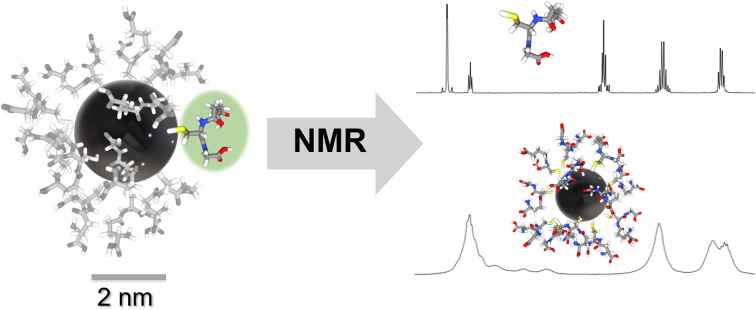

## Introduction

Inorganic nanoparticles, usually stabilized by organic ligands, have attracted considerable interest due to their potential applications in biomedicine^1–5^ and heterogeneous catalysis,^6–11^ including electrocatalysis.^12–15^ It is now well accepted that the ligand shell controls the interaction with the surrounding solvent but also with other nanoparticles or biological molecules, notably the formation of the so-called protein corona in biological media.^16,17^ To understand these interactions, the nature of the ligand shell as well as the surface structure of the nanoparticles need to be elucidated. Here, NMR is one of the major analytical tools. Unfortunately, NMR spectra of nanoparticles in dispersion often lack spectral resolution due to extreme line broadening.^18^ The potential alternative, solid-state NMR spectroscopy on dried particles, requires a considerable amount of material and does not necessarily reflect the solvated state of dispersed nanoparticles.

The situation changes when the nanoparticles become ultrasmall with a diameter of 1–3 nm and below. Such particles have emerged as promising agents in biomedicine^1,2,19–23^ as they are able to penetrate biological barriers and are less prone to form a protein corona.^16,24^ They can also be covalently functionalized with therapeutic agents or targeting moieties.^19,25,26^ In the case of ultrasmall nanoparticles, solution-state NMR has been shown to be applicable to elucidate nature and structure of the ligands and, in favorable cases, to gain insight into the surface structure of the nanoparticle core.^27,28^ In 2015, Marbella and Millstone gave a comprehensive overview on the state of the art, demonstrating how solution-state NMR, solid-state NMR, and metal NMR (*e.g.*^195^Pt) can be applied to nanoparticles.^18^

In this focused review, we concentrate on recent developments and the potential of NMR spectroscopy to analyze the ligand shell of dispersed ultrasmall metal nanoparticles with a size of a few nm. A special emphasis is put on organic ligands which can interact with biomolecules. This is highlighted on examples from our recent research in this area. We will further demonstrate how the surface ligands can be detected and quantified and how this can be used in supramolecular chemistry, *e.g.* to control nanoparticle–biomolecule interactions. We will focus on metallic nanoparticles but also touch metalloid clusters to keep the topic in focus.

### Ultrasmall nanoparticles and atom-sharp metal clusters

The synthesis of ultrasmall nanoparticles and metalloid clusters from solution was developed decades ago.^29–32^ Ultrasmall metal nanoparticles and atom-sharp metal clusters are basically prepared in the same way, *i.e.* by reduction of the corresponding metal salts either in water or in organic solvents, based on the Brust–Schiffrin synthesis^33–35^ which has been modified and refined over time.^36^ The presence of a capping ligand is crucial to avoid particle growth and agglomeration. The most prominent metals are noble metals like gold, silver, and platinum due to their strongly positive reduction potential and their stability towards oxidation, *e.g.* upon dispersion in water. The high specific surface area of ultrasmall nanoparticles makes them sensitive to oxidation.^37^ If they are ultrasmall (1–3 nm) in diameter, they consist of several tens up to several hundreds of metal atoms, with many of them located at the particle surface.^38,39^ A stable conjugation with capping ligands usually occurs *via* soft Lewis acid atoms in the ligands, *i.e.* sulfur or phosphorus.^40^ This makes thiols and phosphanes the preferred ligands. Depending on the way of the synthesis, it is possible to prepare ultrasmall metal nanoparticles (usually on a milligram scale) or atom-sharp metalloid clusters (usually on a smaller scale).^10,41,42^ While atom-sharp metalloid clusters have a defined stoichiometric composition together with a crystal structure with a fixed ratio of metal atoms and ligands, ultrasmall nanoparticles are more disperse in terms of size, shape, and structure.

In general, the size of the capping ligands is of the same order as the nanoparticle core diameter. The number of ligands is typically 30–60% of the number of metal atoms due to the high surface curvature and the high number of metal atoms on the particle surface.^10^ For instance, prominent atom-sharp clusters are Au_25_(SR)_18_ (SR is a thiolated ligand),^43^ Au_32_(R_3_P)_12_Cl_8_ (R = Et, ^n^Pr, ^n^Bu),^44^ Au_102_(pMBA)_44_ (pMBA is *para*-mercapto benzoic acid),^45^ and Au_144_(SR)_60_.^46^ For ligand-coated ultrasmall gold nanoparticles, typical stoichiometries of Au_∼250_GSH_∼125_ (GSH is glutathione),^47^ Au_∼174_(cysteine)_∼67_,^48^ and Au_∼250_(CGSGGGpTPA)_∼150_ (CGSGGGpTPA is a nonapeptide)^49^ were determined by NMR spectroscopy. A comprehensive assessment by NMR spectroscopy together with elemental analysis gave Au_∼250_GSH_∼150_.^50^ Terrill *et al.* found Au_∼400_(SR)_∼127_ (SR is dodecanethiol) by a combination of different methods.^51^

### Limitations of NMR spectroscopy for larger nanoparticles

Solution NMR experiments on ligand-capped metal nanoparticles and clusters mainly focus on the ligand shell and in favorable cases on the surface structure of these nanoparticles. For this, the NMR-active nuclei of the ligands (usually ^1^H and ^13^C, and in some cases ^19^F, ^31^P, and ^15^N) are used as probes. One of the crucial parameters for the interpretation of the spectra is the spectral resolution which is influenced (among many other parameters) by the size of the metal particle. It has been found that the ^1^H and ^13^C NMR linewidths increase with increasing particle diameter^52,53^ and, furthermore, that this line broadening decreases with increasing distance of the corresponding nucleus from the nanoparticle surface. In addition, resonances of ligands are shifted to higher frequencies in case of a coordination on the particle surface.^54^ One is tempted to attribute both results to the fact that the electronic structure of metal particles depends on their size.^38,39^ For example, metallic behavior has been observed for thiol-capped Pt nanoparticles with a diameter of 2.8 nm, Ag particles of 3.0 nm and Pd particles of 5.0 nm, while spheres smaller than 2 nm are usually considered as non-metallic.^55,56^ Recent studies on atom-precise nanoclusters^57,58^ gave a detailed look on this transition; others show that the core electronics can be affected by the surface chemistry and even the solvent.^59,60^ However, calculations and the lacking Korringa relationship of spin-lattice relaxation and temperature indicate that the observed shifts of the ligand signals cannot be attributed to a Knight-shift type mechanism such as in metals.^56,61,62^ Nevertheless, the increase of the paramagnetic contribution to the chemical shift has been assigned to the electronic properties of the metal particles.^63,64^

The broadening of the resonance lines of nuclei close to the surface and its particle size-dependence are commonly understood as a result of the restricted mobility of the nanoparticle and the increasing heterogeneity on the particle surface, *i.e.* the increasing number of binding sites and chemically different environments. With longer distance from the surface, the mobility of the molecules increases and structural inhomogeneities are averaged out.^52,65–67^ In addition, transient magnetic fields causing spin–spin relaxation (*T*_2_ relaxation) must be taken into account as another relaxation pathway resulting in larger ^1^H and ^13^C linewidths for molecules on metallic particles larger than 3 nm.^68^ Therefore, the chances to get direct insight into the surface structure are diminishing at larger particle sizes, but focusing on ultrasmall nanoparticles potentially allows to reach this goal.

Solid-state NMR spectroscopy has been reported for nanoparticles, including heteroatom NMR spectroscopy,^69–73^ but its general applicability suffers from the fact that a considerable amount of substance is required for this experiment. Furthermore, it the ligand dynamics are different in the solid state compared to the solvated dispersed state.

### One-dimensional NMR spectroscopy

^1^H NMR spectroscopy is the first and obvious choice for studying the ligand shell: It offers high sensitivity and the possibility of quantification, *i.e.* to determine the concentration of ligand molecules in the sample. However, the ubiquitous line broadening, the small spectral range and – especially for dilute samples – the need to suppress the solvent signals makes it difficult to get meaningful integration values. Nevertheless, this information can be obtained by addition of an internal standard such as maleic acid^47^ or by applying the ERETIC technique.^74,75^ Both methods should only be used in standard ^1^H experiments without suppression of the solvent signal because these pulse methods are known to distort the signal intensities.^76^ In addition, the concentration of the external or internal standard should be selected so that the signal intensities are comparable to those of the analyzed sample (same order of magnitude).

^13^C NMR spectroscopy faces the opposite problems: the spectral range is favorable and usually the line broadening is moderate. On the other hand, the sensitivity is very low, which often leads to measurement times in the range of hours. Usually, ^13^C NMR spectra are recorded with ^1^H decoupling during data acquisition, which leads to a signal enhancement due to the nuclear Overhauser effect (NOE). The disadvantage of this decoupling mode is that the ^13^C signals cannot be quantified. In contrast, “inverse gated decoupling” allows quantification of the resulting spectra, but requires unacceptably long measurement times.

The number of resonances and the chemical shift of the signals can still give important information about the ligand shell and in some cases about the core–shell-interaction. A less frequently applied technique is ^19^F NMR spectroscopy on fluoride-carrying ligands as shown for an Au_24_ cluster.^77^

A quick ^1^H 1D NMR experiment can often give valuable information whether the ligand attachment to an ultrasmall nanoparticle has been successful and whether the ligand is still intact and not degraded. This information is not easy to obtain for plasmonic nanoparticles, whose NMR spectra have insufficient resolution or do not show NMR signals at all (see below).^18^

### Multidimensional NMR spectroscopy

Given the above mentioned limitations of ^1^H and ^13^C NMR, it is advisable to use the advantages of both methods: The sensitivity of the one and the spectral range of the other in two-dimensional NMR experiments such as ^1^H–^13^C HSQC (Heteronuclear Single Quantum Correlation), ^1^H–^13^C HMQC (Heteronuclear Multiple Quantum Correlation), both yielding ^1^J_CH_ correlations, and ^1^H–^13^C HMBC (Heteronuclear Multiple Bond Correlation over 2–3 bonds) which allow to improve the spectral resolution by expansion into a second dimension and to correlate ^1^H and ^13^C resonances to each other and to the corresponding functional groups.^46,78,79^ In favorable cases, the correlation peaks reveal signals which are difficult to identify in the corresponding one-dimensional spectra.^50^ This was also shown for Pd_55_ clusters, surrounded by dendrimers.^80^

Besides these heteronuclear NMR techniques, homonuclear correlation spectroscopy has been applied in two ways. ^1^H–^1^H COSY (COrrelation SpectroscopY) as well as TOCSY (TOtal Correlation SpectroscopY)^27,54^ provide the connectivity of nuclei within the ligand molecules *via*^n^J-couplings (*n* = 3,4, …). For uniformly labeled compounds, even ^13^C–^13^C bonds were detected by the INADEQUATE technique (Incredible Natural Abundance DoublE QUAntum Transfer Experiment) for ^1^J_CC_ correlations.^48^ Furthermore, the ^1^H–^1^H dipolar interaction has been exploited to extract intermolecular distance information using the nuclear Overhauser effect in NOESY and ROESY experiments.^54,81,82^ For small molecules with a short correlation time *τ*_c_, the NOE is positive, while it turns negative for large molecules (long *τ*_c_). Therefore, for a certain molecule size (depending on the magnetic field), the NOE is zero and does not yield signals in the NOESY spectrum, despite spins being close in space. In this case the less sensitive ROESY experiment offers a good alternative as the phase of the correlation peaks does not change.^83^

### The advantage of isotope-labeled ligands and sensitivity enhancement techniques

The increased sensitivity of high-field spectrometers in combination with cryoprobes enables the investigation of diluted nanoparticle solutions with significantly broadened NMR resonance lines.^48,50,84^ While dynamic nuclear (hyper)polarization (DNP) is more often used in solid state NMR, it can be applied to enhance sensitivity in solution as well.^85,86^ Broadband ^1^H decoupling is often used in 1D ^13^C spectra – not only to collapse splitting due to ^1^H–^13^C *J*-coupling, but also to increase the sensitivity *via* a steady-state heteronuclear NOE.^87^ Lee *et al.* provided a good in-depth review of such sensitivity enhancement methods.^88^

The use of ^13^C isotope-labeled ligands considerably improves the signal-to-noise ratio in 1D ^13^C and ^1^H–^13^C HSQC spectra. For instance, single carbon labelling is available for the thiol-carrying amino acid cysteine which avoids line splitting due to ^13^C–^13^C couplings that occur for cysteine-^13^C_3_ with all three carbon atoms labeled.^48^ Ideally, the labeled ^13^C atom is present as close to the nanoparticle surface as possible, *e.g.* cysteine 3–^13^C with ^13^C at the β-carbon atom that carries the thiol group, to obtain the most detailed information about the ligand–nanoparticle interface. The ^13^C–^13^C INADEQUATE experiment requires unacceptably long experiment times when used on natural abundance samples (1% ^13^C) because it relies on the *J*-coupling between two ^13^C atoms, *i.e.* on one out of 10 000 C–C bonds. Uniform ^13^C-labeling (98–99%) greatly enhances the signal and thus allows for shorter experiment time.

This ^13^C-labeling approach allowed us to identify three different attachment sites for cysteine on the nanoparticle surface.^48^ Recently, a covalent Au–C bond of ^13^C isotope labeled ligands on Au nanoparticles was detected by solid-state NMR spectroscopy,^69^ highlighting its potential in special cases when sufficient sample material is available. It also highlights the potential of selective ^13^C labeling, in particular on positions that are close to the metal surface.

For peptide ligands, ^15^N-labeling allows the observation of the peptide N–H in a ^1^H–^15^N HSQC spectrum, which has proven useful to monitor protein binding to peptide-coated nanoparticles.^49^ Label placement is recommended towards the C-terminus because the N-terminal amino acid (with a free NH_3_^+^ group) and the next 2–4 residues quickly exchange the amide H with solvent H_2_O and are thus invisible, despite isotope labelling.

For unlabeled ligands, the sensitivity of HSQC/HMQC experiments can be increased by fast-acquisition methods like SOFAST-HMQC^89^ or BEST-HSQC.^90^ A selective excitation of the ligand signals (but not the solvent signals) permits much shorter experiments compared to traditional HSQC/HMQC experiments, or, *vice versa*, the collection of 5 up to 20 times more transients in the same time period. Transverse Relaxation Optimized SpectroscopY (TROSY)^91^ can improve sensitivity for large systems by selecting the longest-lived coherences and can be combined with fast pulsing methods, *e.g.* in BEST-TROSY-HSQC.^92^

### NMR techniques to determine the nanoparticle size

The hydrodynamic radius *r*_H_ of a spherical particle is related to its translational diffusion coefficient *D*.^93^ Diffusion Ordered NMR SpectroscopY (DOSY)^94–98^ yields *D* from ^1^H or ^13^C NMR spectra, independent of the density of the particle.^51,52^ DOSY experiments based on 2D ^1^H–^13^C HMQC^99^ or -HSQC spectra^100^ can be used for nanoparticles with ^13^C-labeled ligands like ^13^C-cysteine^48^ or higher concentrated particle dispersions with unlabeled ligands at the natural abundance of the ^13^C isotope.^84^

The signal attenuation *I*/*I*_0_ is measured as a function of a pulsed field gradient *G* where smaller molecules (with larger *D*) experience a steeper decline of signal intensity. To avoid systematic errors, it is important to calibrate the gradient pulses with a sample of known diffusion coefficient (*e.g.* 1% H_2_O in D_2_O) and to ensure stable temperature control. Optimization of the gradient attenuation (gradient pulse length), such that the signal is attenuated to about 10% of its original intensity at the highest gradient strength, provides an optimum coverage of the diffusional decay curve and thus the best reliability of the fitted *D*. Large systems like nanoparticles require relatively long gradient pulses (>2 ms) during which *J*-modulation occurs, which causes negative signals and skews the fitted value of *D*. DOSY experiments incorporating pure shift techniques^101,102^ or the Oneshot45 experiment^103^ suppress these artifacts and yield more accurate diffusion coefficients. Diffusion gradient probes with high gradient powers allow to keep the gradient duration shorter. Proper baseline correction of the spectra is important to obtain correct signal intensities. Close to the solvent signal, especially when suppressed, phase artifacts can occur, therefore signals close to the solvent peak should be excluded. Chemical exchange, *e.g.* exchange of labile protons (–OH, –NH, –SH) with H_2_O, leads to an averaged diffusion coefficient, therefore, signals undergoing exchange should be excluded from analysis as well.

The DOSY plot (*D vs.* chemical shift) allows to distinguish whether different signals belong to molecules of different size. For a reasonably homogeneous nanoparticle population, we prefer the linearized Stejskal–Tanner diagram (ln(*I*/*I*_0_) *vs. G*^2^), where a linear fit gives *D* from the slope.^94^ If two or more particle sizes and/or free ligand are present in solution, their NMR signals mostly overlap. If the diffusion coefficients are similar, the biphasic signal decay cannot be distinguished from a monophasic one.^104^ Therefore, for a given size distribution of particles, an intermediate diffusion coefficient is obtained. For larger differences in *D*, the Stejskal–Tanner plot shows a deviation from linearity and adopts a curved shape. If the difference in *D* is large enough (*e.g.* between nanoparticle and free ligand contamination), one can observe and fit a linear part of the curve at high gradient strengths. For these data points, the signal intensity of the smaller component in the spectrum has decayed below the noise level and only signal from the larger component remains. Free ligand (or ligand disulfide) contamination can often be spotted in the spectrum when sharp signals from the small molecules are overlapped with the broadened signals from particle-bound ligands. The use of an internal standard^105^ yields *r*_H_ from the ratio of the diffusion coefficients and *r*_H_ of the standard, which makes the method more robust towards variations in measuring conditions. The internal standard should ideally exhibit a similar diffusion coefficient as the sample in question, so that the decay curves for both molecules can be resolved in the same experiment with the same gradient settings.

Gomez *et al.* developed a method to determine the nanoparticle core size based on a simple ^1^H NMR experiment, using dendrimer-encapsulated Pd nanoparticles as example:^106^ Ligand protons close to the particle core experience stronger line broadening than protons in the periphery. The ratio of outer to inner proton signal intensities shows a linear correlation with the number of metal atoms in the core particle. Because the intensity ratio from signals within the same ligand are used, this method can be applied independently of the number of ligands per particle. However, the correlation has to be calibrated with several samples of nanoparticles with different and well-defined metal core diameters.

Compared to other methods that probe the particle size, NMR spectroscopy has the advantage that it is performed directly on dispersed nanoparticles, and DOSY gives the hydrodynamic diameter of the particle including the ligand shell. This distinguishes it from small-angle scattering (SAXS) of dispersions where only the inorganic core is probed.^107^ It can be superior to dynamic light scattering (DLS) which sometimes has difficulties with very small particles.^108^ Furthermore, it avoids the intrinsic error of differential centrifugal sedimentation (also known as analytical disc centrifugation) where the hydrodynamic particle diameter is systematically underestimated.^108^ Electron microscopy only works on dried samples and cannot elucidate the diameter of the ligand shell.^109^ However, if the ligands are sufficiently large like proteins, they can be visualized and even quantified by cryo-electron microscopy as Sheibani *et al.*^110^ have shown for the protein corona^111^ on polystyrene nanoparticles.

### The nanoparticle core, the particle surface, and the number of attached ligands

The core of a metallic nanoparticle or a metalloid cluster consists of metal atoms. Depending on the nature of the nanoparticle, this can be either a cut-out of the metal structure (usually fcc, hcp, or bcc) or a more covalent arrangement of the metal atoms.^10,112^ In solution, the core itself is not accessible by NMR spectroscopy because most metal atoms (*e.g.* Au) are not NMR susceptible. In the cases of ^109^Ag and ^195^Pt NMR spectra, even ultrasmall particles yield extremely broad resonances^70^ which severely limits structural interpretation. Transmission electron microscopy (TEM), X-ray powder diffraction (XRD), small-angle X-ray scattering (SAXS), and X-ray photoelectron spectroscopy (XPS) are more suitable tools to characterize the metal core and its internal structure, but they are not very sensitive to the nature of the nanoparticle surface structure. This is where NMR spectroscopy of ultrasmall nanoparticles comes into play. By analyzing the structure and chemical shift of the attached ligands, it is possible to elucidate the nature of their interaction with the particle surface. It can even be used to discriminate chiral nanoparticles by the identification of diastereotopic protons of attached ligands.^113^

The quantification of the number of ligand molecules is also possible *via* NMR spectroscopy. This requires the ligand concentration, the nanoparticle concentration, and the nanoparticle diameter (on case of a sphere) and gives the molecular footprint, *i.e.* the area covered (or needed) by each ligand on the nanoparticle surface. The molecular footprint is often surprisingly small with 0.1 nm^2^ per ligand or less,^37,50,67,114^ indicating a high density of ligands on the particle surface facilitated by the high surface curvature of the ultrasmall nanoparticles.^53^ Wu *et al.* have quantified the ligand shell consisting of (16-mercaptohexadecyl)trimethylammonium bromide on gold nanoparticles from 1.2 to 25 nm by quantitative ^1^H NMR spectroscopy both in dispersion and after reductive detachment of the ligands. In general, the number of ligands was lower if quantified after detachment compared to signal integration of the very broad NMR peaks. They found molecular footprints of about 0.2 nm^2^ for gold particles smaller than 10 nm and about 0.3 nm^2^ for gold particles of 25 nm.^53^ Terrill *et al.* found 0.14 nm^2^ for 127 dodecanethiol molecules on one 2.4 nm gold nanoparticle.^51^ This is obviously lower than the typical value for thiols on flat gold (111) surfaces (0.22 nm^2^) as reported earlier.^115,116^

**Fig. 1:**
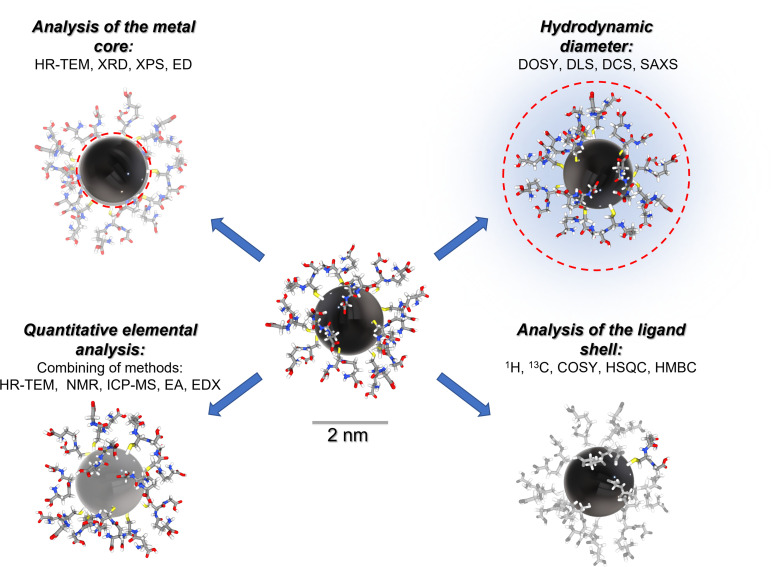
Comprehensive analysis of an ultrasmall nanoparticle by a variety of analytical methods. HR-TEM: high-resolution transmission electron microscopy; XRD: X-ray diffraction; XPS: X-ray photoelectron spectroscopy; ED: electron diffraction; DOSY: diffusion-ordered spectroscopy; DLS: dynamic light scattering; DCS: differential centrifugal sedimentation; SAXS: small-angle X-ray scattering; ICP-MS: inductively coupled plasma mass spectrometry; EA: elemental analysis; EDX: energy-dispersive X-ray spectroscopy.

Fig. 1 gives a schematic representation of a comprehensive characterization of an ultrasmall nanoparticle by a variety of analytical methods.

In the following, we will illustrate the potential of NMR spectroscopy with four examples of ultrasmall metal nanoparticles.

#### Example 1: cysteine-coated gold nanoparticles

**Fig. 2:**
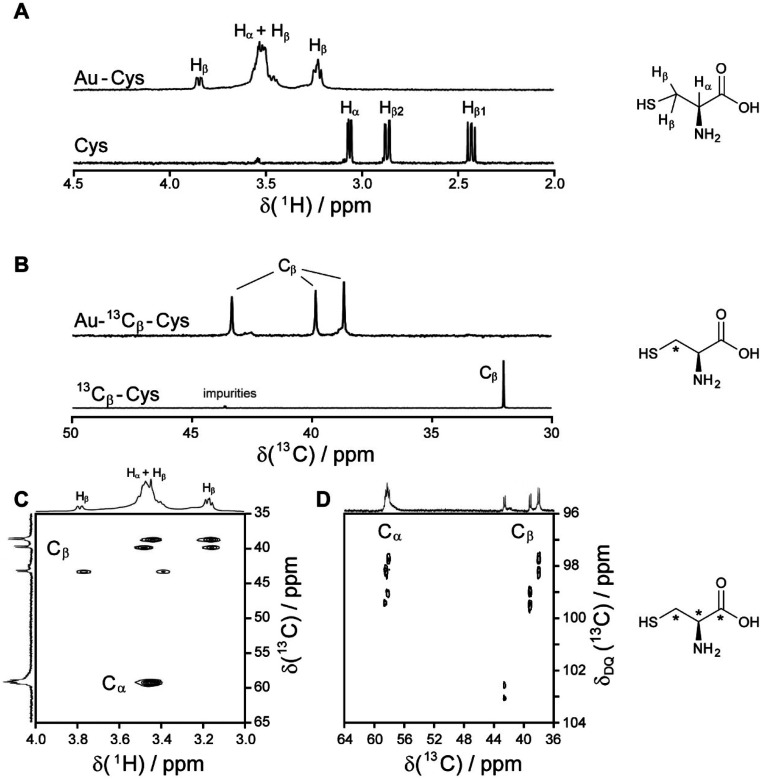
NMR spectra of cysteine-coated ultrasmall gold nanoparticles (Au-Cys). The chemical structure of cysteine is shown on the right, and ^13^C-labeled atoms are marked with an asterisk. (A) ^1^H NMR spectrum of Au-Cys (top) and dissolved cysteine (bottom) in H_2_O (pH 12). The signals of Au-bound cysteine are shifted downfield, broadened, and H_β_ signals are split and partially overlapping with the H_α_ signals. (B) ^13^C NMR spectra of ^13^C_β_-labeled Au-Cys (top) and dissolved ^13^C_β_-cysteine (bottom). The C_β_ signals are split into three distinct peaks, indicating three different chemical environments on the nanoparticle surface. (C) The ^1^H–^13^C HSQC spectrum of fully ^13^C-labeled Au–^13^C-Cys shows three sets of H_β_/C_β_ cross-peaks while the Ha/Ca signals overlap. (D) The ^13^C–^13^C INADEQUATE spectrum of Au–^13^C-Cys correlates each C atom to its neighbors, linking each of the three C_β_ atoms to a different C_α_ atom (C_α_ signals of the C_β_ atom at 42 ppm are not visible; *δ*_DQ_ is the sum of chemical shifts of the observed atom and its neighbors). This figure has been adapted from ref. 48 with permission from the American Chemical Society, copyright 2019.

l-Cysteine is the only naturally occurring amino acid with a thiol group. Therefore, it is of special interest as ligand for ultrasmall metallic nanoparticles because it allows the attachment of peptides and potentially proteins. We have conducted a thorough NMR-spectroscopic characterization of water-dispersed cysteine-coated gold nanoparticles to elucidate the arrangement of the cysteine ligands on the gold surface.^48^ The ^1^H NMR spectrum of gold-bound cysteine shows strong line broadening in addition to the paramagnetic shift due to the metal surface (Fig. 2), as it has been frequently reported for ligands bound to ultrasmall nanoparticles.^66,78,113,117,118^ The ^1^H COSY and ^1^H NOESY spectra revealed a complex coupling pattern and signal overlap, rendering the ^1^H spectra alone insufficient to unambiguously assign all signals. Thus, ^13^C (both fully and ^13^C_β_-only) and ^15^N isotope labelling became essential for the interpretation of the ligand spectra.

Interestingly, three distinct signals of the cysteine C_β_ atom (at 39 ppm, 40 ppm and 43 ppm) were observed in the ^13^C NMR spectrum. In the ^1^H–^13^C HSQC spectrum, these three C_β_ species coupled with three distinct sets of diastereotopic H_β_ protons. Similarly, the amino group showed three signals in the ^15^N NMR spectrum. All these C_β_ and amino group signals experienced a shift to higher frequencies compared to the C_β_ signal of free cysteine, consistent with being bound to gold, and thus indicating three different chemical environments for cysteine on the gold surface. The C_α_ atoms of these three cysteine species only gave rise to one broad H_α_/C_α_ signal in the ^1^H–^13^C HSQC spectrum but could be distinguished as three independent signals in the C_α_/C_β_ coupling region of the ^13^C–^13^C INADEQUATE spectrum. ^13^C DOSY and ^1^H–^13^C iDOSY-HSQC diffusion experiments showed that all three cysteine species were stably bound to particles of the same size. We attributed this to cysteine ligands bound to three different crystal faces.^112^ Binding sites with different chemical environments, either due to different crystal faces or chirality of the ligand arrangement on the nanoparticle surface, have been reported for other ligands like glutathione (GSH),^84,119^ up to the distinction of 22 different sites in Au_102_(pMBA)_44_ gold cluster with *para*-mercaptobenzoic acid (pMBA) as ligand.^54^

#### Example 2: glutathione-stabilized nanoparticles of different metals

The tripeptide glutathione (GSH) consisting of glutamate, cysteine and glycine has been introduced in 1996 to stabilize gold clusters Au_*x*_(GSH)_*y*_ of different sizes.^35,120^ It has now emerged as a kind of model ligand for metallic nanoparticles.^121–124^ It forms a stable shell around gold nanoparticles,^47,78,125^ but also around silver and platinum nanoparticles^50,84^ as well as nanoparticles of platinum group metal oxides.^37^ The stability of the M–S bond allows a further covalent functionalization. For example, the conversion of amine groups to azide groups opens a way to attach bioactive compounds or dyes to the ligand shell by the well-established copper-catalyzed azide–alkyne cycloaddition.^47^

**Fig. 3:**
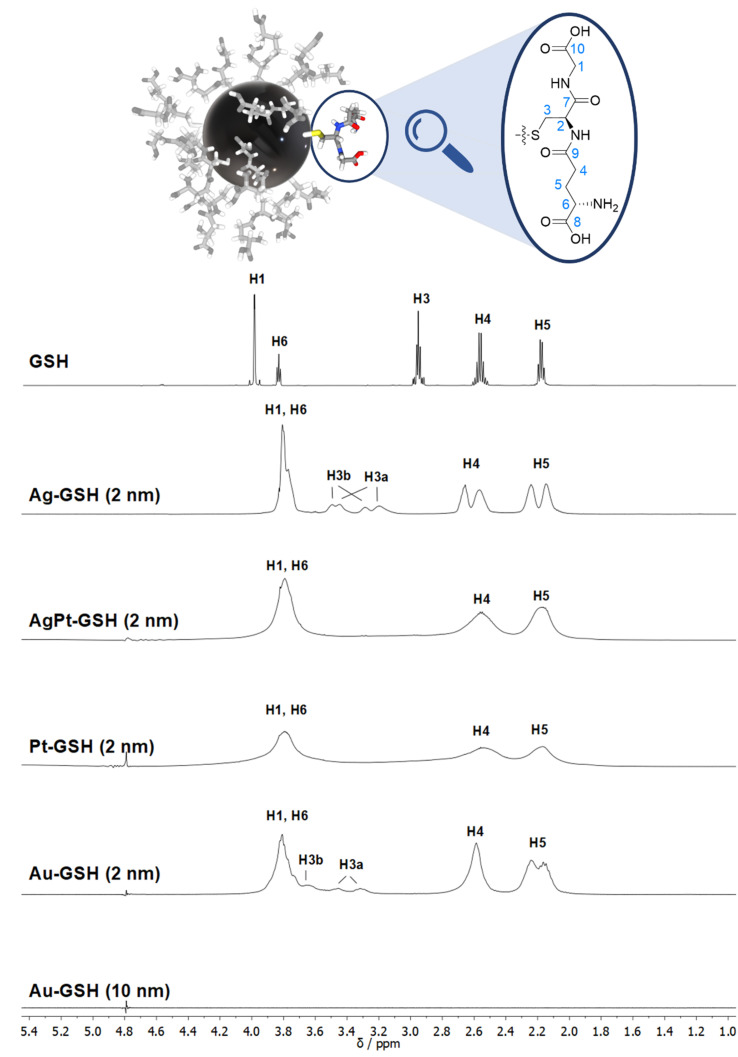
Comparison of the ^1^H NMR spectra of different metal nanoparticles (2 nm) and a plasmonic gold nanoparticle (10 nm), all stabilized with glutathione (GSH).^50^ Note the strong line broadening in the presence of platinum and the complete absence of all NMR signals for the larger gold nanoparticles (10 nm). The solvent water was suppressed (see the small signal at 4.79 ppm). The image on the top shows a 2 nm gold nanoparticle with glutathione ligands drawn to scale. This figure has been adapted from ref. 50 with permission from the American Chemical Society, copyright 2023.

The NMR spectrum of an ultrasmall nanoparticle depends on its size as well as on the metal core. Fig. 3 shows a comparison of different metal nanoparticles with 2 nm diameter and a plasmonic gold nanoparticle (10 nm). Cores of gold and silver give moderately broadened spectra whereas the presence of platinum causes strong line broadening, also if present as an alloy with silver (50 : 50 mol%).^50^ Notably, there are no signals of the ligand for plasmonic gold nanoparticles with 10 nm diameter as shown in the spectrum in Fig. 3, underscoring that larger (plasmonic) nanoparticles completely evade NMR spectroscopy. However, the complete cancellation of the NMR signal in the vicinity of plasmonic nanoparticles can be used to compute the concentration of adsorbed proteins in an indirect way as shown by Xu *et al.*^126^ The integral of the NMR signal intensity represents the fraction of non-adsorbed (=free) protein. Thus, the decrease in NMR signal intensity is proportional to the adsorbed fraction where the NMR intensity is canceled. In a similar way, Gomez *et al.* have demonstrated how the size of a palladium core (55 to 250 Pd atoms) coated by a dendrimer can be determined by comparing the ^1^H-NMR intensity as a function of the distance from the metal core (see above).^106^

While many ligands establish an Au–S bond *via* their terminal thiol group, GSH is anchored on the central amino acid (cysteine). Although a detailed NMR investigation of the interaction between GSH and even ultrasmall metal particles is limited by the omnipresent line broadening of all ^1^H and ^13^C signals, the application of the full NMR toolbox allows deep insights.

**Fig. 4:**
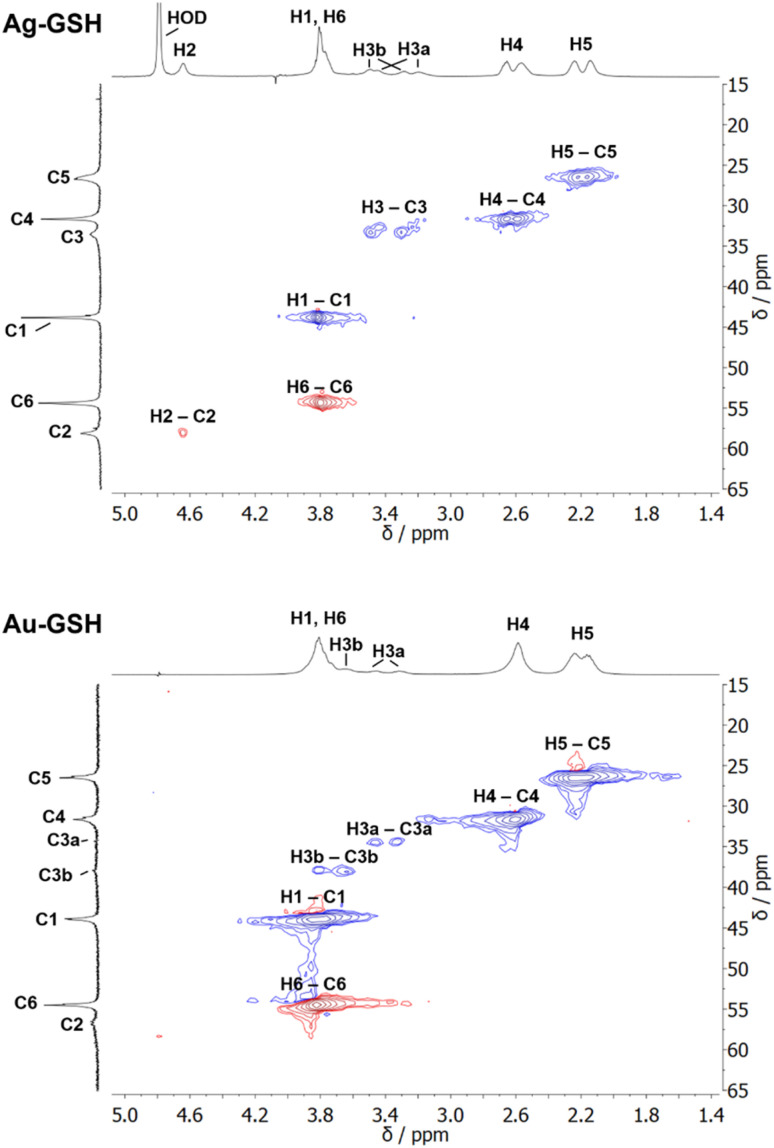
^1^H–^13^C HSQC NMR spectra of ultrasmall GSH-stabilized nanoparticles (99.9% D_2_O; pH 8.5). For gold nanoparticles, a clear assignment of H3a/C3a and H3b/C3b peaks was possible, indicating two different positions of glutathione on the nanoparticle surface. For silver nanoparticles, the resolution was significantly lower, but a second ^13^C resonance was detected earlier.^84^ The colors indicate the different types of carbon atoms as determined by the phase: blue: CH_2_; red: CH, CH_3_. This figure has been adapted from ref. 50 with permission from the American Chemical Society, copyright 2023.

^1^H NMR spectra of these water-dispersed nanoparticles usually require the suppression of the solvent signal (trace amounts of HOD in D_2_O or H_2_O/D_2_O mixtures), resulting in a better observability of most of the signals except those close to 4.79 ppm such as the signal of H2 (Fig. 3). Nevertheless, in comparison with the ^1^H NMR spectrum of a GSH solution (at the same pH value), the signals can be assigned. Of special interest is the β-CH_2_ group (C3 and H3) of the cysteine residue in glutathione which is close to the metal surface. Most interestingly, in some cases not only two signals of the diastereotopic H3 protons are observed; instead, in the NMR spectrum of silver nanoparticles, four well-resolved signals for H3 were detected. Three H3 signals were observed for gold nanoparticles, while a fourth signal overlaps with those of H1 and H6 (Fig. 3). However, this only becomes clear when the corresponding ^1^H–^13^C HSQC spectrum (Fig. 4) are carefully analyzed. ^1^H–^1^H COSY spectra generally suffer from low spectral resolution and rarely provide additional information. The value of the ^1^H–^13^C HSQC experiment becomes even clearer when looking at the ^13^C NMR spectra, which can be extremely time-consuming to record. Furthermore, the resonances of the C3 carbon atoms are significantly broader than the other ones making these signals hard to detect. In the two-dimensional experiment, however, the existence of two distinct ^13^C resonances was proven beyond doubt. The HMBC experiment is significantly less sensitive than the HSQC; therefore, correlations of H3 protons with neighboring carbon atoms could not be detected. At least, the signals of the carbonyl carbon atoms C9, C9, and C10 were unambiguously assigned due to their ^3^J spin–spin interaction with the protons H1, H4, and H6.

The detection of different ^1^H and ^13^C signals for the cysteine β-CH_2_ group of glutathione (C3/H3) indicates the presence of two different crystallographic sites for GSH on the metal surface, leading to different magnetic environments and therefore different chemical shifts (as in the case of cysteine).

#### Example 3: peptide-functionalized gold nanoparticles to target the surface of proteins

The recognition of protein surfaces with the aim to modulate protein function is currently a highly investigated area of supramolecular chemistry. Ultrasmall nanoparticles presenting ligands that recognize specific protein epitopes have shown high potential in this respect. We investigated peptide-coated nanoparticles^49^ carrying the phosphothreonine-proline (pTP)^127^ recognition motif for the WW domain of the human protein Pin1 as a proof-of-concept. Pin1 is a peptidyl-prolyl *cis*/*trans* isomerase that regulates cell proliferation and survival.

**Fig. 5:**
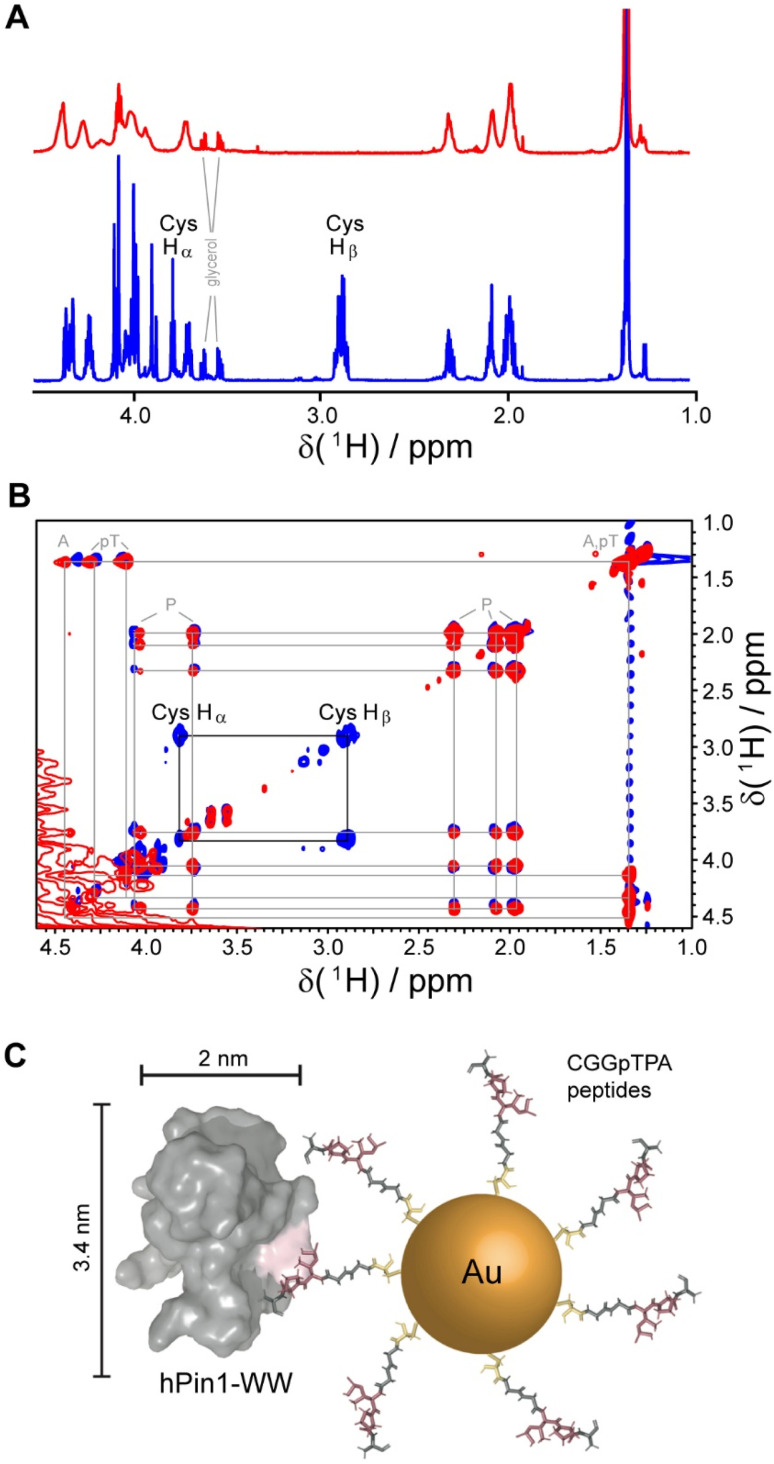
NMR spectroscopic analysis of Au-CGGpTPA peptide-coated ultrasmall gold nanoparticles. (A) ^1^H NMR spectra of Au-CGGpTPA (top, red) and dissolved peptide CGGpTPA (bottom, blue). (B) ^1^H,^1^H TOCSY spectra of Au-CGGpTPA (red) and dissolved peptide CGGpTPA (blue). Cysteine H_α_ and H_β_ signals are not visible for peptides bound to the nanoparticle. (C) A peptide-coated gold nanoparticle and an hPin1-WW protein drawn to scale. This figure has been adapted from ref. 49 with permission from the American Chemical Society, copyright 2021.

Gold nanoparticles were coated with two peptides, *i.e.* either CGGpTPA or CGSGGGpTPA, containing the pTP motif at different distances to their N-terminal cysteine residue which served as attachment point to the gold surface. Nanoparticle-bound peptides lacked the characteristic cysteine H_β_ or H_α_/H_β_ correlation in the ^1^H and ^1^H TOCSY NMR spectra, probably because they were broadened beyond detection (Fig. 5). However, we cannot exclude the possibility that the cysteine H_α_ and H_β_ signals were shifted downfield such that they now overlapped with the H_α_ resonances of the other amino acids, which experience less broadening due to their larger distance from the gold core and increased internal flexibility. DOSY-NMR confirmed that the peptides were bound to the nanoparticles, yielding a hydrodynamic diameter of 4.4 to 5.4 nm, depending on the peptide length.^49^

For quantitative protein binding experiments, it is imperative to know the overall concentration of peptides in the sample, and thus the number of peptides per nanoparticle. Peptide concentrations were determined by quantitative ^1^H NMR spectroscopy, using the methyl group signals of alanine and phosphothreonine with maleic acid as external standard. Together with the gold concentration determined by atomic absorption spectroscopy (AAS) and the average particle core diameter of 2 nm by electron microscopy, about 150 peptide molecules per nanoparticle were calculated, in good agreement with the number determined from UV-Vis spectra of particles coated with the corresponding fluorophore-labeled peptide. Binding constants between the protein and the nanoparticles in comparison to the free peptides were determined by NMR titrations with ^15^N-labeled protein and by isothermal titration calorimetry (ITC). Protein binding to the nanoparticles showed an apparently decreased affinity if a 1 : 1 stoichiometry was assumed. However, given the size of the particles and the protein, it is obvious for steric reasons that not each peptide can bind to a protein. Geometrically, we estimated that about 20 proteins fit around one Au-CGGpTPA particle. Taking this into account by introducing a stoichiometric factor into the fit, both ITC and NMR yielded about 18 proteins binding to each nanoparticle, in good agreement with the geometric estimation.^49^

#### Example 4: at the resolution limit: silver-glutathione nanoparticles, functionalized with a fluorescent dye

**Fig. 6:**
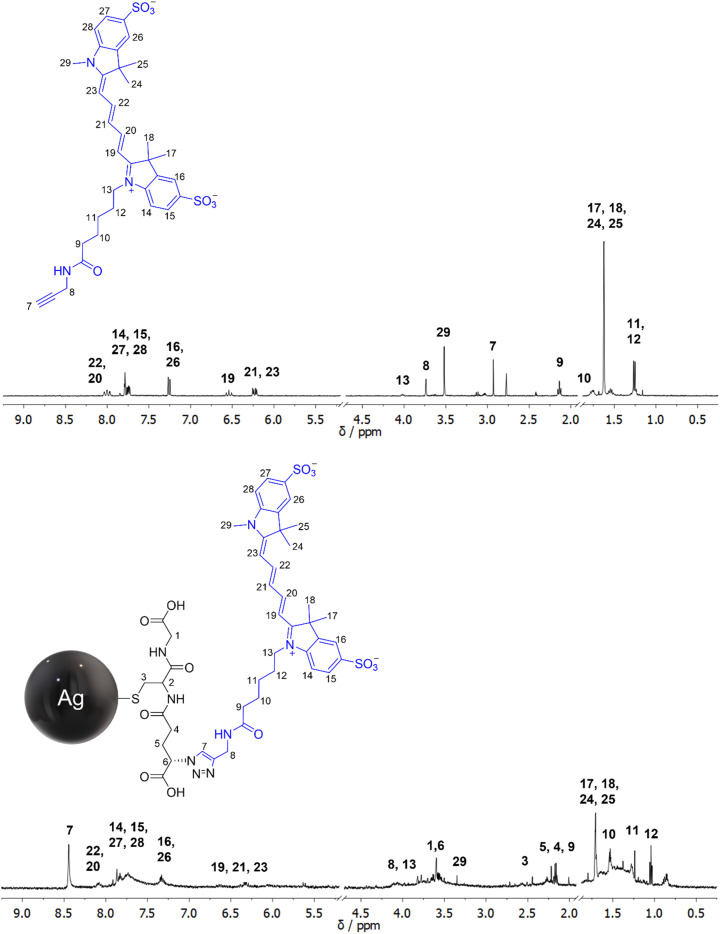
^1^H NMR spectra of the dissolved (free) alkyne-terminated dye sulfo-Cy5 (top) and the dye clicked to glutathione-azide-terminated silver nanoparticles (bottom). The spectrum of sulfo-Cy5 (blue) on the nanoparticles is rather crowded due to inherent peak broadening and the presence of the primary ligand glutathione (black). Thus, the peak assignments are uncertain in most cases. However, the alkyne proton of the dissolved dye (H7 at 2.93 ppm) and the triazole proton of the clicked dye (H7 at 8.44 ppm) are clearly visible, confirming the successful click reaction. The spectra were recorded at 600 MHz in 90% H_2_O/10% D_2_O at pH 8.0. The water suppression signal was cut out (4.79 ppm). In the bottom spectrum, a large solvent peak at 1.90 ppm was cut out.

It is possible to attach organic fluorescent dyes to the surface of azide-terminated metal nanoparticles *via* click chemistry. For this, the amine groups of glutathione ligands on the metal surface are converted into azide groups. Then, an alkyne-terminated dye is attached.^47^ The NMR signals of the dye can be clearly seen in the NMR spectrum. Notably, the NMR signals of nuclei which are farther away from the metal surface are less broadened than those in closer vicinity (*e.g.* the aromatic and olefine protons of sulfo-Cy5-alkyne). However, the inherent peak shift and peak broadening make the assignment of the NMR signals difficult. Fig. 6 gives an illustrative example where the dye sulfo-Cy5-alkyne was clicked to glutathione-azide-terminated silver nanoparticles. The assignment of the ^1^H NMR peaks is difficult and not always possible with sufficient confidence. The overlap of the peaks makes their integration and quantification difficult. Furthermore, the signals of the primary ligand glutathione show up as well. In general, it is easier to quantify complex ligands by UV spectroscopy than by NMR spectroscopy if they have a reasonably strong peak in the UV spectrum which is the case for all fluorescent dyes. UV spectroscopy also works for nucleic acids that have a characteristic UV absorption.^128^ This illustrates the limit of NMR spectroscopy if very complex ligands are attached.

## Conclusions

As shown, NMR spectroscopy has a strong potential to elucidate the nature of ultrasmall nanoparticles and atom-sharp clusters. The value of this technique is obvious although there are limitations to be aware of. In the following, we sum up what can and what cannot be accomplished by NMR spectroscopy on nanoparticles.

(1) The spectral resolution depends on the particle size and the core metal. Larger nanoparticles give rise to broad resonances in the NMR spectrum approaching the detection limit. Thus, they may be easily overlooked. Consequently, NMR spectroscopy cannot be used to prove the absence of larger particles or agglomerates. In contrast, free dissolved ligands can be easily distinguished from bound ligands due to the difference in their linewidths and chemical shifts. It is therefore recommended to record well-resolved NMR spectra of the dissolved ligands and to record all NMR spectra at the same pH (if water is used as solvent).

(2) NMR spectroscopy has an inherently low sensitivity that becomes critical in the case of nanoparticles. However, state-of-the-art hardware, like high-field spectrometers with cryoprobes, in combination with sensitivity-enhancing and fast-acquisition pulse sequences push the sensitivity limit. In addition, isotope enrichment is particularly valuable for ^13^C NMR spectra. It selectively or uniformly enhances the signal intensity and makes it possible to distinguish between individual carbon atoms. This is important when the colloidal stability of a dispersion of nanoparticles is insufficient to achieve the concentration required for NMR spectroscopy.

(3) NMR signals are strongly broadened in the vicinity of a metal particle. Furthermore, the line widths within an NMR spectrum often vary greatly even for one ligand which makes their separation difficult. This can lead to very complex spectra where a qualitative analysis may range from difficult to fully impossible. Here, two-dimensional NMR spectroscopy often helps to solve this problem. By a combination of homo- and heteronuclear techniques, NMR peaks can be assigned and sometimes even quantified.

(4) The number of ligands on one nanoparticle can be determined by quantitative ^1^H NMR spectroscopy. This is achieved either with an external standard or by the ERETIC method. In both cases, a reliable integration of isolated signals is a prerequisite. Furthermore, the concentration of nanoparticles in the dispersion must be known.

That being said, we conclude that NMR spectroscopy on dispersed ultrasmall nanoparticles can also help to elucidate subsequent chemical reaction steps on the particle surface as it is sensitive to type and number of attached ligands. Furthermore, it permits to study the interaction between nanoparticles and other molecules, *e.g.* proteins. Quantitative interaction parameters like *K*_D_ values can be derived. This opens a new area in supramolecular chemistry, *e.g.* to study ligand–protein interactions, which provides more specific information than less structure-specific techniques like isothermal titration calorimetry or small-angle X-ray scattering.

## Conflicts of interest

There are no conflicts to declare.
